# Investigating the macular choriocapillaris in early primary open-angle glaucoma using swept-source optical coherence tomography angiography

**DOI:** 10.3389/fmed.2022.999167

**Published:** 2022-09-21

**Authors:** Katherine Lun, Yin Ci Sim, Rachel Chong, Damon Wong, Bingyao Tan, Rahat Husain, Tin Aung, Chelvin C. A. Sng, Leopold Schmetterer, Jacqueline Chua

**Affiliations:** ^1^Department of Ophthalmology, National University Health System, Singapore, Singapore; ^2^Yong Loo Lin School of Medicine, National University of Singapore, Singapore, Singapore; ^3^Singapore National Eye Centre, Singapore Eye Research Institute, Singapore, Singapore; ^4^Ophthalmology & Visual Sciences Academic Clinical Program, Duke-NUS Medical School, Singapore, Singapore; ^5^SERI-NTU Advanced Ocular Engineering (STANCE), Singapore, Singapore; ^6^School of Chemical and Biomedical Engineering, Nanyang Technological University, Singapore, Singapore; ^7^Institute of Molecular and Clinical Ophthalmology, Basel, Switzerland; ^8^Department of Clinical Pharmacology, Medical University Vienna, Vienna, Austria; ^9^Center for Medical Physics and Biomedical Engineering, Medical University Vienna, Vienna, Austria

**Keywords:** primary open-angle glaucoma, choroid, choriocapillaris, swept-source optical coherence tomography angiography, glaucoma

## Abstract

**Introduction:**

There has been a growing interest in the role of vascular factors in glaucoma. Studies have looked at the characteristics of macular choriocapillaris in patients with glaucoma but with conflicting results. Our study aims to use swept-source optical coherence tomography angiography (SS-OCTA) to evaluate macular choriocapillaris metrics in normal participants and compare them with patients with early primary open-angle glaucoma (POAG) (mean deviation better than −6dB).

**Methods:**

In this prospective, observational, cross-sectional study, 104 normal controls (157 eyes) and 100 patients with POAG (144 eyes) underwent 3 mm × 3mm imaging of the macula using the Plex Elite 9000 (Zeiss Meditec, Dublin, CA, USA). Choriocapillaris OCTA images were extracted from the device’s built-in review software and were subsequently evaluated for the density and size of choriocapillaris flow deficits.

**Results:**

After adjusting for confounding factors, the density of flow deficits was independently higher in those aged 53 years and above (*P* ≤ 0.024) whereas the average flow deficit size was significantly larger in those aged 69 years and above (95% CI = 12.39 to 72.91; *P* = 0.006) in both normal and POAG patients. There were no significant differences in the density of flow deficits (*P* = 0.453) and average flow deficit size (*P* = 0.637) between normal and POAG participants.

**Conclusion:**

Our study found that macular choriocapillaris microvasculature on SS-OCTA is unaltered by subjects with POAG. This suggests that OCTA macular choriocapillaris may not be potentially helpful in differentiating early glaucoma from healthy eyes.

## Introduction

Glaucoma is an optic neuropathy associated with progressive loss of retinal ganglion cells and their axons, with resultant structural changes at the optic nerve head (ONH) (1). The ONH is believed to be the primary site of damage in glaucoma and disruption of its blood flow and the surrounding peripapillary retina is believed to play a role in its pathogenesis (2, 3). Deeper structures of the ONH, such as the lamina cribrosa, and the choroid, share the same blood supply (posterior ciliary artery) (4, 5), and various studies have reported abnormal choroidal blood flow parameters in patients with glaucoma (6–10). Evaluation of choroidal hemodynamics was challenging with previous imaging modalities such as fluorescein angiography (11), indocyanine green angiography (12), and laser doppler flowmetry (9). This was due to the invasive nature of tests (6), an inability to differentiate choroidal vascular layers (6, 9, 13), and the inability to have reproducible, quantitative measurements (13). Fortunately, with the arrival of optical coherence tomography angiography (OCTA) (14–16), a non-invasive imaging modality that allows the quantitative assessment of the microcirculation of the choroid, vascular layers of the choroid can be better examined and it has become possible to assess macular choriocapillaris circulation in patients with glaucoma. In addition, in cases where evaluation of the ONH is challenging due to anatomical features of the optic disc, OCTA may serve as an additional diagnostic tool to detect early glaucoma by assessing the disruptions of macular choriocapillaris in these patients.

Studies examining the macular choroidal circulation using OCTA are, unfortunately, limited with conflicting results in subjects with glaucoma (17–20). Chao et al. used spectral domain optical coherence tomography angiography (SD-OCTA; Angiovue, Optovue Inc., Bayview, CA, USA) to evaluate macular circulation in patients with glaucoma [18 eyes with open angle glaucoma, 14 with normal tension glaucoma (NTG), ocular hypertension (OHT) (18 eyes)] and healthy subjects and did not find any difference in choriocapillaris perfusion between groups (17). Similarly, Milani et al. examined healthy individuals and patients with POAG (39 eyes) and OHT (43 eyes) using SD-OCTA (XR Avanti device with the AngioVue imaging system) and did not find any significant differences in macular choriocapillaris flow perfusion area between groups (18). On the other hand, Yip et al. carried out a cross-sectional study on healthy subjects and glaucoma subjects (15 eyes with POAG, 14 with NTG, 1 Juvenile open angle glaucoma, and 2 eyes with angle closure glaucoma) using SD-OCTA (XR Avanti with Angiovue imaging system and novel in-house developed software to determine vessel density) and found a reduction in microvascular density of the macula and optic disc in glaucoma patients compared with healthy controls (20). Lastly, Tepelus et al. used swept source (SS)-OCTA (Plex Elite 9000, Zeiss Meditec, Dublin, CA, USA) and reported lower choriocapillaris perfusion density in NTG patients (49 eyes) when compared to normal subjects (40 eyes) (19). It is not clear whether the variations seen in these studies were due to imaging modality differences (SS-OCTA vs. SD-OCTA) or the discrepancies in study design (small sample size of < 35 subjects), pathological subgroup (i.e., POAG, NTG, OHT), and analytical method (frequency matching by age or statistical adjustments of confounding factors such as age, glaucoma severity, and signal strength), thus making direct comparisons between normal and glaucoma eyes challenging.

Therefore, we evaluated the macular choriocapillaris metrics using SS-OCTA Plex Elite 9000 in healthy participants and individuals having early primary open-angle glaucoma (POAG). Clinically, there is an interest to detect glaucoma in the earlier stages to enable timely treatment and to minimize the risk of irreversible visual field loss. Hence OCTA may act as an additional diagnostic tool that can assess damage to the macular choriocapillaris vasculature in glaucoma patients.

## Materials and methods

### Participants

In this prospective cross-sectional study, participants aged 21 years and older (21–99 years old) were consecutively recruited from the Singapore National Eye Centre, a tertiary eye care institution in Singapore, between July 2018 to May 2021. This study was approved by the SingHealth Centralized Institutional Review Board, Singapore (protocol number R1500/83/2017) and conducted in accordance with the Declaration of Helsinki, with written informed consent obtained from all participants.

Patients with early primary open-angle glaucoma (POAG) patients were defined by the following criteria during an ophthalmic examination: presence of glaucomatous optic neuropathy (defined as loss of neuroretinal rim with a vertical cup: disc ratio of > 0.7 or an inter-eye asymmetry of > 0.2 and/or notching attributable to glaucoma) with compatible and reproducible visual fields in standard automated perimetry (glaucoma hemifield test outside normal limits) with mean deviation (MD) better than -6dB (21), open angles on gonioscopy, and absence of secondary causes of glaucomatous optic neuropathy (22, 23). Normal controls were individuals who did not have clinically relevant eye conditions, such as glaucoma, age-related macular degeneration, diabetic retinopathy, and ocular vascular occlusive disorders, diabetes and other causes of neuro-ophthalmic disease (24). POAG patients were on the following intra-ocular pressure lowing eye drops: prostaglandin analogs (latanoprost, bimatoprost, travoprost, tafluprost), beta blockers (timolol), alpha-2 adrenergic agonists (brimonidine), and carbonic anhydrase inhibitors (brinzolamide).

### Ocular examinations

Participants underwent auto-refraction-keratometry (Canon RK-5 Autorefractor Keratometer; Canon Inc., Tokyo, Japan) and intra-ocular pressure measurement using airpuff tonometer at the Singapore Eye Research Institute. Spherical equivalent was calculated as the spherical value plus half of the negative cylinder value. Central corneal thickness was measured using an ultrasound pachymeter (Advent; Mentor O & O Inc., Norwell, MA, USA); the mean of the five measurements were used for analysis (25).

Demographic data, medical history (e.g., diabetes and systemic hypertension), ocular history (e.g., eye diseases) and medication use were collected from all participants using a detailed interviewer-administered questionnaire. A digital automatic blood pressure monitor (Dinamap model Pro Series DP110X-RW, 100V2; GE Medical Systems Information Technologies, Inc., Milwaukee, WI, USA) was used to measure systolic and diastolic blood pressures (SBP, DBP) after subjects were seated for at least 5 min (26). Blood pressure was measured twice, 5 min apart. If the previous 2 SBP readings differed by more than 10 mmHg or the DBP by more than 5 mmHg, a third measurement was then taken.

### Imaging acquisition

During the same visit, participants underwent 3 mm × 3mm macular-centered imaging using SS-OCTA (Plex Elite 9000; Version 1.7; Carl Zeiss Meditec). Both eyes of each participant were imaged after pharmacological dilation (Tropicamide 1%). To ensure high quality images were taken, all images were obtained by a trained ophthalmic photographer (HQ) and acquisitions were repeated multiple times. Each scan consisted of four repeated volumes of 300 cross-sectional images, and each image consisted of 300 A-scans (27).

### Imaging analysis

Quality of OCTA scans were reviewed by one trained grader who was masked to the participant’s characteristics. Poor quality scans were defined as having any of the following characteristics: (i) poor signal strength (index < 6), (ii) poor clarity (i.e., blurred vessels), (iii) significant motion artifacts visible as irregular vessel patterns on the en-face angiogram, (iv) segmentation error, or (v) local weak signal caused by artifacts such as floaters (28). Both eyes were included in the study only if both met the eligibility criteria.

**FIGURE 1:**
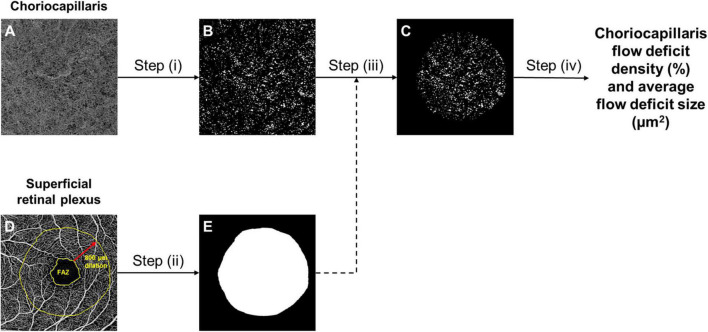
The framework of choriocapillaris OCTA image processing. Raw choriocapillaris OCTA image extracted from the OCTA machine **(A)** was binarized with a threshold of 1.5 standard deviation below the mean intensity of the image to segment flow deficits **(B)**. Foveal avascular zone (FAZ) segmented from the raw superficial retinal plexus OCTA image was dilated by 800 μm **(D)** to generate a mask **(E)** that indicates the region to be analysed. The mask **(E)** was applied to the segmented flow deficits **(B)** to generate the final flow deficit image **(C)** where the density and size of flow deficits were computed.

Choriocapillaris OCTA images, spanning from 31 μm below the retinal pigment epithelium (RPE) to 40 μm below the RPE, were extracted from the built-in review software (Carl Zeiss Meditec, Inc., Dublin, CA, USA) (29). These OCTA images were subsequently loaded into a customized MATLAB (The MathWorks Inc., Natick, MA, USA) algorithm that evaluates the density of flow deficits in the choriocapillaris automatically (30). The algorithm comprises of the following steps (Figure 1): (i) binarization of flow deficits in the choriocapillaris OCTA image by setting a threshold that is 1.5 standard deviation below the mean intensity of the image; (ii) dilation of the foveal avascular zone (FAZ; segmented *via* a trained U-Net prior to analysis) in the superficial retinal plexus by 800 μm to generate a mask that indicates the region to be analyzed; (iii) application of the mask on the binarized flow deficit image; (iv) computation of choriocapillaris flow deficit density as the percentage of flow deficit area per total imaged area in the region of interest, and flow deficit size (μm^2^) as the total flow deficit area divided by the total number of flow deficits in the region of interest.

### Statistical analyses

Primary outcome was the density and size of choriocapillaris flow deficits. Shapiro–Wilk test was used to assess the normality of the distribution of the continuous variables. We compared the variables between groups using one-way analysis of variance (ANOVA) for normally distributed continuous variables or Kruskal–Wallis equality-of-populations rank test for non-normally distributed continuous variables and with Chi-square tests or Fisher’s exact tests for categorical variables. We determined the strength of the correlation between choriocapillaris flow deficits and blood pressure using Pearson correlation coefficient, where *r* value less than 0.3, between 0.3 and 0.5, and greater than 0.50 indicate small, moderate, and strong correlation, respectively (31). To analyze correlated eye data, multivariable linear regression analysis with generalized estimating equations (GEE) was performed to assess the effect of age (performed only in normal controls) and eye diseases (independent variables) on density or size of the choriocapillaris flow deficit (dependent variable), adjusting for potential confounders such as diabetes, hypertension, intraocular pressure, axial length, and signal strength of scans. Since the recruited patients come from an ongoing, existing study consisting of glaucoma patients and normal controls, we did a *post hoc* power calculation to evaluate the statistical power of the existing study (*n* = 100 glaucoma cases vs. 104 controls) using the means and standard deviations derived from the current study. For choriocapillaris density (9.06 ± 0.14% vs. 8.90 ± 0.13%), using an alpha error of 5%, we would have a *post hoc* power of 100%. For size, using 283 ± 5 μm^2^ vs. 287 ± 7 μm^2^, we would have a *post hoc* power of 100%.^1^ *P*-value < 0.05 was considered statistically significant. Data were analyzed with statistical software (STATA, version 16; StataCorp LP).

## Results

Of the 235 participants recruited for the study, 31 (13.2%) were excluded because of poor quality OCTA images. This left 104 normal controls and 100 glaucoma subjects for analysis. The median (interquartile range) age was 59.0 (11.5) years for normal controls and 62.0 (12.0) years for glaucoma patients. Patients with glaucoma had significantly lower intraocular pressure, longer axial length, and scans of lower signal strength (Table 1). Glaucoma patients were also more likely to have diabetes and hypertension than normal controls (*P* < 0.001).

**TABLE 1:** Comparison of demographics, systemic, and ocular characteristics between normal control and primary open angle glaucoma patients.

	Normal control	Early POAG	*P-value
**Number of participants**	104	100	
Age, years	59.0 (11.5)	62.0 (12.0)	0.134
Gender, Male	51 (49.0)	53 (53.0)	0.572
Ethnicity, Chinese	91 (87.5)	87 (87.0)	0.287
Diabetes	0 (0)	24 (24.0)	**< 0.001**
Hypertension	2 (1.9)	40 (40.0)	**< 0.001**
Systolic blood pressure, mmHg	136.3 (18.6)	129.9 (25.7)	0.093
Diastolic blood pressure, mmHg	76.5 (14.6)	74.8 (14.8)	0.339
**Number of eyes**	157	144	
Intraocular pressure, mmHg	17 (5)	14 (4)	**< 0.001**
Axial length, mm	24.11 (1.71)	24.81 (2.17)	**< 0.001**
Visual field mean deviation (MD), dB	–	−2.40 (1.66)	–
Signal strength of scans^†^	9.15 ± 0.64	8.85 ± 0.93	**0.004**

Data are number (%), mean ± standard deviation (SD), or median (interquartile range), as appropriate.

*Test for differences between groups, based on one-way analysis of variance (ANOVA) for normally distributed continuous variables or Kruskal–Wallis equality-of-populations rank test for non-normally distributed continuous variables and with Chi-square tests or Fisher’s exact tests for categorical variables.

^†^1 represents poor scan quality while 10 represents high scan quality. dB, decibels; POAG, primary open angle glaucoma. Bold values denote statistical significance at the P < 0.05 level.

There was marginal positive correlation between choriocapillaris characteristics and systolic blood pressure (density: *r* = 0.050, *P* = 0.411; size: *r* = 0.007, P = 0.898). Among the POAG patients, 12% (12 patients) were not on any form of glaucoma medications (five were post-cataract and trabeculectomy surgery, two were patients with stable NTG and not on treatment, two had poor adherence to medications and were not using medications at point of recruitment, and three were not started on medications yet). For the remaining 88%, 57% were on one medication, and 31% were on two or more types. Amongst the patients using glaucoma medications, 75% were using prostaglandin analogues, 25% beta blockers, 18% alpha-2 adrenergic agonists, and 7% carbonic anhydrase inhibitors. Neither number of glaucoma medications nor types of glaucoma medications were associated with choriocapillaris characteristics (*P* ≥ 0.05).

**FIGURE 2:**
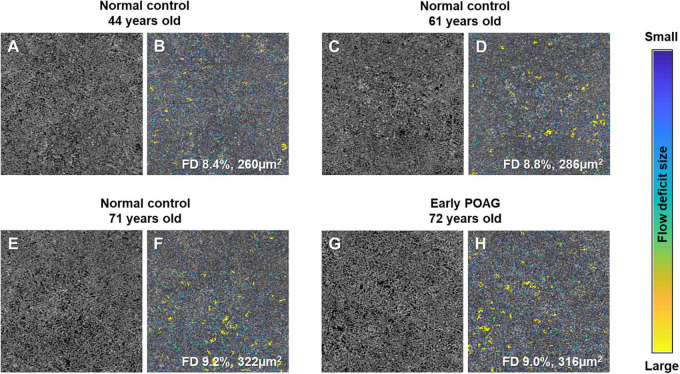
Representative choriocapillaris OCTA 3 × 3 mm2 images **(A,C,E,G)** and their corresponding color-coded maps **(B,D,F,H)**. The density and average size of choriocapillaris flow deficits (FD) in normal controls increases with age, where it is 8.4% and 260 μm^2^ in the 44-year-old subject **(A,B)**, followed by 8.8% and 286 μm^2^ in the 61-year-old subject **(C,D)**, and highest at 9.2% and 322 μm^2^ in the 71-year-old **(E,F)**. Similar density and average size of choriocapillaris FD can be seen in a 72-year-old patient with mild primary open angle glaucoma (POAG; visual field mean deviation of −2.85 dB; G and H; 9.0% and 316 μm^2^) and a 71-year-old normal control **(E,F**; 9.2% and 322 μm^2^).

**FIGURE 3:**
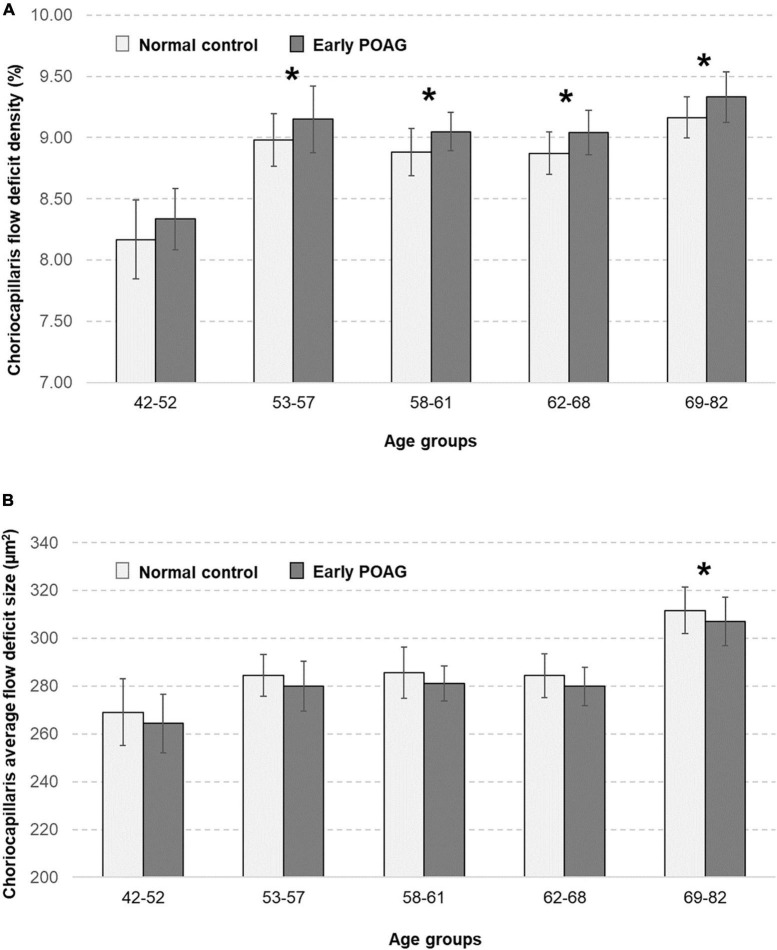
Relationship between the choriocapillaris **(A)** flow deficit density (%) and **(B)** average flow deficit size (μm^2^) in normal controls (light grey bars) and patients with early primary open angle glaucoma (POAG; dark grey bars), stratified by age groups. Choriocapillaris flow deficit density was evidently higher in those aged 53 years and above (all *P* ≤ 0.024) whereas the average flow deficit size was significantly larger in those aged 69 years and above (*P* = 0.006) in both normal and glaucoma patients. There were no differences in the choriocapillaris characteristics between normal and glaucoma patients. Asterisks mark significant differences in reference to the youngest age group according to multivariable linear regression analysis with generalized estimating equations (GEE) adjusted for diabetes, hypertension, intraocular pressure, axial length, and signal strength of scans.

The multivariable linear regression modeling of associations of choriocapillaris density and size with normal aging and glaucoma, while controlling for diabetes, hypertension, intraocular pressure, axial length, and signal strength of scans are as shown in Table 2. Persons who were in the older age groups (53–82 years old) tended to have more flow deficits as compared to those in the youngest age group (*P* ≤ 0.24). In terms of the average flow deficit size, it was significantly larger in the oldest age group (*P* < 0.001) whereas it appeared similar for those aged 53–68 years old (*P* ≥ 0.237) when compared to the youngest group (42–52 years old). Specifically, the oldest group (69–82 years old) had 1% higher density of flow deficits in the choriocapillaris (95% CI = 0.34 to 1.65; *P* = 0.003) that were also 42.65 μm^2^ larger in size (95% CI = 12.39 to 72.91; *P* = 0.006) than those in the youngest age group. In contrast, the density (*P* = 0.453) and size (*P* = 0.637) of flow deficits in the choriocapillaris were similar between POAG and normal participants. Figure 2 shows representative OCTA images taken from normal controls of different age groups and glaucoma patients and illustrates the above findings, highlighting the increasing size of choriocapillaris flow deficit density with age. There were no differences in the density and size of choriocapillaris flow deficit between POAG patients and normal controls. Figure 3 is a graphical representation of the general increment of choriocapillaris flow deficits in terms of its density and size with age in both glaucoma patients and normal controls. There was a statistically significant difference in choriocapillaris flow deficit density in patients aged 53 years and older compared to the reference age group (all *P* ≤ 0.024). When comparing the average flow deficit size, the average flow deficit size was significantly larger only in those aged 69 years and above (*P* = 0.006) in both normal controls and glaucoma patients.

**TABLE 2:** Multivariable linear regression modeling of the association between normal aging, primary open angle glaucoma and choriocapillaris flow deficits.

	Flow deficit density (%)			Average flow deficit size (μ m^2^)		
		
Characteristic	β	95% CI	*P-value	β	95% CI	*P-value
**Normal aging**						
Age quintile, years						
42–52 (*n* = 35 eyes of 22 patients)		Reference			Reference	
53–57 (*n* = 36 eyes of 23 patients)	0.81	0.13 to 1.49	**0.019**	15.52	−13.30 to 44.33	0.291
58–61 (*n* = 32 eyes of 19 patients)	0.71	0.16 to 1.27	**0.012**	16.66	−10.93 to 44.25	0.237
62–68 (*n* = 31 eyes of 20 patients)	0.71	0.09 to 1.32	**0.024**	15.45	−12.59 to 44.47	0.280
69–82 (*n* = 23 eyes of 20 patients)	1.00	0.34 to 1.65	**0.003**	42.65	12.39 to 72.91	**0.006**
**Eye diseases**						
Normal (*n* = 157 eyes of 104 patients)		Reference			Reference	
Early POAG (*n* = 144 of 100 patients)	0.17	−0.27 to 0.60	0.453	−4.56	−23.60 to 14.44	0.637

*Adjusted for diabetes, hypertension, intraocular pressure, axial length, and signal strength of scans. Bold values denote statistical significance at the P < 0.05 level. β, beta coefficient; CI, confidence interval; NA, not applicable; POAG, primary open angle glaucoma. Bold values denote statistical significance at the P < 0.05 level.

## Discussion

In our study, we used SS-OCTA to examine macular choriocapillaris in normal and early POAG participants. After adjusting for relevant confounding factors such as diabetes, hypertension, intraocular pressure, axial length, and signal strength of scans, we found that older patients were more likely to have less perfused choriocapillaris (e.g., larger sized flow deficits) as compared to younger patients. In contrast, we did not find any significant differences in macular choriocapillaris features between normal and early POAG participants.

Ours is the largest study to date demonstrating that flow patterns in the macular choriocapillaris is not altered in early glaucoma. The current study had a statistical power of 100% to detect a minimal difference of 0.17% for density and −4.56 μm^2^ for size, between glaucoma cases and normal controls. Previous smaller studies (with ≤ 35 subjects in each group) either found a difference (20) in choriocapillaris in glaucoma subjects, or none (17, 18). When small sample size is used, the study may have low statistical power and hence carry a risk that observations occur due to chance. Our study is adequately powered with 104 normal controls and 100 glaucoma patients. There was a tendency for a difference between glaucoma patients and normal controls at all ages (Figure 3). It is likely that this difference could become significant as OCTA technology advances and higher quality images can be obtained. On the other hand, while larger studies detect tiny or small associations, these findings may not be clinically important or relevant in improving the detection of early glaucoma.

Apart from the small sample size, earlier studies did not account for relevant confounding factors (32), such as axial length (17) and signal strength of scans (17, 18, 20) which are well-known to affect OCTA metrics. Another potential discrepancy is the severity of glaucoma as the differences in choriocapillaris flow deficits may be more prominent in more advanced glaucoma. In the paper by Yip et al. (20) the mean deviation of glaucoma subjects was −11.07 (±8.25) dB, suggesting that the study may have had patients with more moderate-severe glaucoma, whereas our POAG participants had early glaucoma (visual field mean deviation score of −2.40 (± −1.66) dB.

By allowing in-depth assessment of the choroidal circulation in a non-invasive manner, SS-OCTA has improved our understanding of ocular circulation and its role in the pathogenesis of eye diseases, including glaucoma (14, 15, 33). The use of SS-OCTA to study choriocapillaris hemodynamics seems to be more advantageous compared to the use of SD- OCTA in previous studies (17, 18, 20). Compared to SD-OCTA, SS-OCTA uses a longer wavelength (1050 nm) which allows deeper penetration and enhanced imaging of choroidal structures (33). For a given acquisition time, the faster image acquisition of SS-OCTA also enables scan patterns to be denser and of a larger area compared with SD-OCT scans (34, 35). One possible explanation as to why the macular choriocapillaris is unaffected in early POAG is that glaucoma is characterized by the progressive loss of retinal ganglion cells (RGCs), and these cells receive their blood supply from the superficial vascular complex (36) whereas the choriocapillaris supplies the outer retina. On the other hand, in the SS-OCTA study by Tepelus et al. (19) involving 22 NTG patients, eyes with NTG demonstrated lower macular choriocapillaris flow deficit density compared to normal eyes. Unlike POAG, where IOP is the main risk factor, progression of NTG is multifactorial and not solely IOP dependent (37). The vascular theory behind NTG offers a possible explanation for this difference in findings between the two groups. Vascular factors have been hypothesized to contribute to the development and progression of glaucoma (2). It is believed that these factors are especially significant in NTG patients, where optic nerve damage is believed to be a result of vascular dysregulation and poor blood supply (16, 38–40).

Our finding on the impact of normal aging on the macular choriocapillaris is in line with previous studies (41–45). Cheng et al. (41) found that a higher density of choriocapillaris flow deficits was associated with older age among 830 healthy Chinese individuals who were imaged using SS-OCTA. This was also reported by Zheng et al. (42) where the density increased with age, with greatest increase seen in the central 1 mm region of the macula. Similarly, Fujiwara et al. (43) reported a significant negative relationship between vascular density of the choroid and subjects’ age in 163 healthy volunteers. These findings are also consistent with histopathological studies by Ramrattan et al. (46) who showed decreased choriocapillaris density with age. The reason for these age-dependent changes of the choriocapillaris, however, is still not clear. The age-related loss of choriocapillaris flow deficit features should be carefully considered when estimating disease-related choriocapillaris changes.

### Strengths and limitations

The strengths of our study include sufficient study sample size of normal and early POAG participants, use of the SS-OCTA device, and accounting of a comprehensive list of potential confounding factors. Conversely, we recognize the limitations of our study. Our study did not include other glaucoma subtypes. It will be useful to study macular choriocapillaris differences between NTG and POAG patients, especially given the role of vascular factors in NTG pathogenesis as discussed above. Also, our study did not include moderate-severe glaucoma given our intention was to assess whether the OCTA-based vascular metrics of the macular choriocapillaris may offer an additional diagnostic tool to discriminate early glaucoma from normal controls.

## Conclusion

In conclusion, the macular choriocapillaris density with SS-OCTA is affected by normal aging but unaffected by early POAG. Our findings suggest that the macular choriocapillaris perfusion appear to be unaffected by POAG mechanism and may not be a helpful OCTA diagnostic option for early glaucoma.

## Data availability statement

The raw data supporting the conclusions of this article will be made available by the authors, without undue reservation.

## Ethics statement

The studies involving human participants were reviewed and approved by the SingHealth Centralized Institutional Review Board, Singapore (protocol number: R1500/83/2017). The patients/participants provided their written informed consent to participate in this study.

## Author contributions

JC, KL, and LS conceived and designed the study and wrote the main manuscript text. JC, KL, YS, RC, DW, BT, RH, TA, CS, and LS analyzed and interpreted the data. All authors reviewed the manuscript.

## Footnotes

1
https://clincalc.com/stats/Power.aspx

## Abbreviations

ANOVA: analysis of variance
DBP: diastolic blood pressure
GEE: generalized estimating equations
NTG: normal tension glaucoma
OCTA: optical coherence tomography angiography
ONH: optic nerve head
POAG: primary open-angle glaucoma
RPE: retinal pigment epithelium
SD-OCTA: spectral domain optical coherence tomography angiography
SBP: Systolic blood pressure
SS-OCTA: swept-source optical coherence tomography angiography.

## References

[1] Weinreb RN Aung T Medeiros FA. The pathophysiology and treatment of glaucoma: a review. *JAMA.* (2014) 311:1901–11. 10.1001/jama.2014.3192 24825645 PMC4523637

[2] Flammer J Orgul S Costa VP Orzalesi N Krieglstein GK Serra LM The impact of ocular blood flow in glaucoma. *Prog Retin Eye Res.* (2002) 21:359–93. 10.1016/S1350-9462(02)00008-3 12150988

[3] Cherecheanu AP Garhofer G Schmidl D Werkmeister R Schmetterer L. Ocular perfusion pressure and ocular blood flow in glaucoma. *Curr Opin pharmacol.* (2013) 13:36–42. 10.1016/j.coph.2012.09.003 23009741 PMC3553552

[4] Hayreh SS. Blood supply of the optic nerve head. *Ophthalmologica.* (1996) 210:285–95. 10.1159/000310727 8878212

[5] Lejoyeux R Benillouche J Ong J Errera MH Rossi EA Singh SR Choriocapillaris: Fundamentals and advancements. *Prog Retin Eye Res.* (2022) 87:100997. 10.1016/j.preteyeres.2021.100997 34293477

[6] Duijm HF van den Berg TJ Greve EL. Choroidal haemodynamics in glaucoma. *Br J Ophthalmol.* (1997) 81:735–42. 10.1136/bjo.81.9.735 9422924 PMC1722313

[7] Garhofer G Fuchsjager-Mayrl G Vass C Pemp B Hommer A Schmetterer L. Retrobulbar blood flow velocities in open angle glaucoma and their association with mean arterial blood pressure. *Invest Ophthalmol Vis Sci.* (2010) 51:6652–7. 10.1167/iovs.10-5490 20688735

[8] Portmann N Gugleta K Kochkorov A Polunina A Flammer J Orgul S. Choroidal blood flow response to isometric exercise in glaucoma patients and patients with ocular hypertension. *Invest Ophthalmol Vis Sci.* (2011) 52:7068–73. 10.1167/iovs.11-7758 21828157

[9] Marangoni D Falsini B Colotto A Salgarello T Anselmi G Fadda A Subfoveal choroidal blood flow and central retinal function in early glaucoma. *Acta Ophthalmol.* (2012) 90:e288–94. 10.1111/j.1755-3768.2011.02340.x 22268459

[10] Shin DY Hong KE Lee NY Park CK Park HYL. Association of choroidal blood flow with autonomic dysfunction in patients with normal tension glaucoma. *Sci Rep.* (2022) 12:5136. 10.1038/s41598-022-09162-4 35332217 PMC8948179

[11] Yamazaki S Inoue Y Yoshikawa K. Peripapillary fluorescein angiographic findings in primary open angle glaucoma. *Br J Ophthalmol.* (1996) 80:812–7. 10.1136/bjo.80.9.812 8942378 PMC505618

[12] O’Brart DP de Souza Lima M Bartsch DU Freeman W Weinreb RN. Indocyanine green angiography of the peripapillary region in glaucomatous eyes by confocal scanning laser ophthalmoscopy. *Am J Ophthalmol.* (1997) 123:657–66. 10.1016/S0002-9394(14)71078-5 9152071

[13] Kim K Yang J Feuer W Gregori G Kim ES Rosenfeld PJ A comparison study of polypoidal choroidal vasculopathy imaged with indocyanine green angiography and swept-source optical coherence tomography angiography. *Am J Ophthalmol.* (2020) 217:240–51. 10.1016/j.ajo.2020.05.017 32445699

[14] Spaide RF Fujimoto JG Waheed NK Sadda SR Staurenghi G. Optical coherence tomography angiography. *Prog Retin Eye Res.* (2018) 64:1–55. 10.1016/j.preteyeres.2017.11.003 29229445 PMC6404988

[15] Van Melkebeke L Barbosa-Breda J Huygens M Stalmans I. Optical coherence tomography angiography in glaucoma: A review. *Ophthalmic Res.* (2018) 60:1–13. 10.1159/000488495 29794471

[16] Chua J Tan B Ang M Nongpiur ME Tan AC Najjar RP Future clinical applicability of optical coherence tomography angiography. *Clin Exp Optom.* (2019) 102:260–9. 10.1111/cxo.12854 30537233

[17] Chao SC Yang SJ Chen HC Sun CC Liu CH Lee CY. Early macular angiography among patients with glaucoma, ocular hypertension, and normal subjects. *J Ophthalmol.* (2019) 2019:7419470. 10.1155/2019/7419470 30766730 PMC6350555

[18] Milani P Urbini LE Bulone E Nava U Visintin D Cremonesi G The macular choriocapillaris flow in glaucoma and within-day fluctuations: an optical coherence tomography angiography study. *Invest Ophthalmol Vis Sci.* (2021) 62:22. 10.1167/iovs.62.1.22 33475691 PMC7817881

[19] Tepelus TC Song S Borrelli E Nittala MG Baghdasaryan E Sadda SR Quantitative analysis of retinal and choroidal vascular parameters in patients with low tension glaucoma. *J Glaucoma.* (2019) 28:557–62. 10.1097/IJG.0000000000001242 30889061

[20] Yip VCH Wong HT Yong VKY Lim BA Hee OK Cheng J Optical coherence tomography angiography of optic disc and macula vessel density in glaucoma and healthy eyes. *J Glaucoma.* (2019) 28:80–7. 10.1097/IJG.0000000000001125 30461553

[21] Chua J Baskaran M Ong PG Zheng Y Wong TY Aung T Prevalence, risk factors, and visual features of undiagnosed glaucoma: the singapore epidemiology of eye diseases study. *JAMA Ophthalmol.* (2015) 133:938–46. 10.1001/jamaophthalmol.2015.1478 26043441

[22] Chua J Tan B Ke M Schwarzhans F Vass C Wong D Diagnostic ability of individual macular layers by spectral-domain oct in different stages of glaucoma. *Ophthalmol Glaucoma.* (2020) 3:314–26. 10.1016/j.ogla.2020.04.003 32980035

[23] Chua J Schwarzhans F Wong D Li C Husain R Crowston JG Multivariate normative comparison, a novel method for improved use of retinal nerve fiber layer thickness to detect early glaucoma. *Ophthalmol Glaucoma.* (2021) 5:359–68. 10.1016/j.ogla.2021.10.013 34718222

[24] Chua J Tham YC Tan B Devarajan K Schwarzhans F Gan A Age-related changes of individual macular retinal layers among Asians. *Sci Rep.* (2019) 9:20352. 10.1038/s41598-019-56996-6 31889143 PMC6937292

[25] Chua J Tham YC Liao J Zheng Y Aung T Wong TY Ethnic differences of intraocular pressure and central corneal thickness: the Singapore Epidemiology of Eye Diseases study. *Ophthalmology.* (2014) 121:2013–22. 10.1016/j.ophtha.2014.04.041 24950592

[26] Chua J Le TT Tan B Ke M Li C Wong DWK Choriocapillaris microvasculature dysfunction in systemic hypertension. *Sci Rep.* (2021) 11:4603. 10.1038/s41598-021-84136-6 33633311 PMC7907127

[27] Borrelli E Toto L Viggiano P Evangelista F Palmieri M Mastropasqua R. Widefield topographical analysis of the retinal perfusion and neuroretinal thickness in healthy eyes: a pilot study. *Eye (Lond).* (2020) 34:2264–70. 10.1038/s41433-020-0804-5 32055020 PMC7784843

[28] Hong J Tan B Quang ND Gupta P Lin E Wong D Intra-session repeatability of quantitative metrics using widefield optical coherence tomography angiography (OCTA) in elderly subjects. *Acta Ophthalmol.* (2019) 98:570–8. 10.1111/aos.14327 31833241 PMC7496426

[29] Lin E Ke M Tan B Yao X Wong D Ong L Are choriocapillaris flow void features robust to diurnal variations? A swept-source optical coherence tomography angiography (OCTA) study. *Sci Rep.* (2020) 10:11249. 10.1038/s41598-020-68204-x 32647298 PMC7347889

[30] Zhang Q Zheng F Motulsky EH Gregori G Chu Z Chen CL A Novel Strategy for Quantifying Choriocapillaris Flow Voids Using Swept-Source OCT Angiography. *Invest Ophthalmol Vis Sci.* (2018) 59:203–11. 10.1167/iovs.17-22953 29340648 PMC5770182

[31] Cohen J. Set Correlation and Contingency Tables. *Appl Psychol Meas.* (1988) 12:425–34. 10.1177/014662168801200410

[32] Chua J Sim R Tan B Wong D Yao X Liu X Optical coherence tomography angiography in diabetes and diabetic retinopathy. *J Clin Med.* (2020) 9:1723. 10.3390/jcm9061723 32503234 PMC7357089

[33] Lains I Wang JC Cui Y Katz R Vingopoulos F Staurenghi G Retinal applications of swept source optical coherence tomography (OCT) and optical coherence tomography angiography (OCTA). *Prog Retin Eye Res.* (2021) 84:100951. 10.1016/j.preteyeres.2021.100951 33516833

[34] Novais EA Adhi M Moult EM Louzada RN Cole ED Husvogt L Choroidal neovascularization analyzed on ultrahigh-speed swept-source optical coherence tomography angiography compared to spectral-domain optical coherence tomography angiography. *Am J Ophthalmol.* (2016) 164:80–8. 10.1016/j.ajo.2016.01.011 26851725 PMC4811690

[35] Miller AR Roisman L Zhang Q Zheng F Rafael de Oliveira Dias J Yehoshua Z Comparison between spectral-domain and swept-source optical coherence tomography angiographic imaging of choroidal neovascularization. *Invest Ophthalmol Vis Sci.* (2017) 58:1499–505. 10.1167/iovs.16-20969 28273316 PMC5361583

[36] Penteado RC Zangwill LM Daga FB Saunders LJ Manalastas PIC Shoji T Optical coherence tomography angiography macular vascular density measurements and the central 10-2 visual field in glaucoma. *J Glaucoma.* (2018) 27:481–9. 10.1097/IJG.0000000000000964 29664832 PMC5986603

[37] Anderson DR. Normal tension glaucoma s. collaborative normal tension glaucoma study. *Curr Opin Ophthalmol.* (2003) 14:86–90. 10.1097/00055735-200304000-00006 12698048

[38] Nakazawa T. Ocular blood flow and influencing factors for glaucoma. *Asia Pac J Ophthalmol (Phila).* (2016) 5:38–44. 10.1097/APO.0000000000000183 26886118

[39] Schmidl D Garhofer G Schmetterer L. The complex interaction between ocular perfusion pressure and ocular blood flow - relevance for glaucoma. *Exp Eye Res.* (2011) 93:141–55. 10.1016/j.exer.2010.09.002 20868686

[40] Kiyota N Shiga Y Takahashi N Yasuda M Omodaka K Tsuda S Progression in open-angle glaucoma with myopic disc and blood flow in the optic nerve head and peripapillary chorioretinal atrophy zone. *Ophthalmology Glaucoma.* (2020) 3:202–9. 10.1016/j.ogla.2020.03.005 32672617

[41] Cheng W Song Y Lin F Jin L Wang Z Jonas JB Choriocapillaris flow deficits in normal chinese imaged by swept-source optical coherence tomographic angiography. *Am J Ophthalmol.* (2022) 235:143–53. 10.1016/j.ajo.2021.09.018 34582767

[42] Zheng F Zhang Q Shi Y Russell JF Motulsky EH Banta JT Age-dependent changes in the macular choriocapillaris of normal eyes imaged with swept-source optical coherence tomography angiography. *Am J Ophthalmol.* (2019) 200:110–22. 10.1016/j.ajo.2018.12.025 30639367 PMC6513331

[43] Fujiwara A Morizane Y Hosokawa M Kimura S Kumase F Shiode Y Factors affecting choroidal vascular density in normal eyes: quantification using en face swept-source optical coherence tomography. *Am J Ophthalmol.* (2016) 170:1–9. 10.1016/j.ajo.2016.07.006 27430684

[44] Park SH Cho H Hwang SJ Jeon B Seong M Yeom H Changes in the retinal microvasculature measured using optical coherence tomography angiography according to age. *J Clin Med.* (2020) 9:883. 10.3390/jcm9030883 32213852 PMC7141499

[45] Uji A Balasubramanian S Lei J Baghdasaryan E Al-Sheikh M Sadda SR. Choriocapillaris imaging using multiple en face optical coherence tomography angiography image averaging. *JAMA Ophthalmol.* (2017) 135:1197–204. 10.1001/jamaophthalmol.2017.3904 28983552 PMC5710392

[46] Ramrattan RS van der Schaft TL Mooy CM de Bruijn WC Mulder PG de Jong PT. Morphometric analysis of Bruch’s membrane, the choriocapillaris, and the choroid in aging. *Invest Ophthalmol Vis Sci.* (1994) 35:2857–64. 8188481

